# Microbiome–gut–brain dysfunction in prodromal and symptomatic Lewy body diseases

**DOI:** 10.1007/s00415-022-11461-9

**Published:** 2022-11-10

**Authors:** Sephira Ryman, Andrei A. Vakhtin, Sarah Pirio Richardson, Henry C. Lin

**Affiliations:** 1 The Mind Research Network, 1101 Yale Blvd. NE, Albuquerque, NM 87106 USA; 2 Nene and Jamie Koch Comprehensive Movement Disorder Center, Department of Neurology, The University of New Mexico, Albuquerque, NM 87131 USA; 3 Department of Medicine, The University of New Mexico, Albuquerque, NM 87131 USA; 4 Section of Gastroenterology, New Mexico VA Health Care System, Albuquerque, NM 87108 USA

**Keywords:** Idiopathic REM sleep behavior disorder, Parkinson’s disease, Dementia with Lewy bodies, Gut–brain axis, Dysbiosis, Intestinal permeability

## Abstract

Lewy body diseases, such as Parkinson’s disease and dementia with Lewy bodies, vary in their clinical phenotype but exhibit the same defining pathological feature, α-synuclein aggregation. Microbiome–gut–brain dysfunction may play a role in the initiation or progression of disease processes, though there are multiple potential mechanisms. We discuss the need to evaluate gastrointestinal mechanisms of pathogenesis across Lewy body diseases, as disease mechanisms likely span across diagnostic categories and a ‘body first’ clinical syndrome may better account for the heterogeneity of clinical presentations across the disorders. We discuss two primary hypotheses that suggest that either α-synuclein aggregation occurs in the gut and spreads in a prion-like fashion to the brain or systemic inflammatory processes driven by gastrointestinal dysfunction contribute to the pathophysiology of Lewy body diseases. Both of these hypotheses posit that dysbiosis and intestinal permeability are key mechanisms and potential treatment targets. Ultimately, this work can identify early interventions targeting initial disease pathogenic processes before the development of overt motor and cognitive symptoms.

## Introduction

The spectrum of Lewy body diseases ranges from incidental Lewy body disease, Parkinson’s disease (PD) with varying degrees of cognitive impairment, to dementia with Lewy bodies (DLB) at the most severe end [1]. PD is characterized by motor symptoms (bradykinesia, rigidity, and tremor [2]), with recent, increased appreciation that non-motor symptoms are common and significantly impact quality of life [3]. Estimated prevalence of PD ranges between 100 and 200 per 100,000 people and an annual incidence of 15 per 100,000 [4]. DLB is characterized by fluctuating cognition, recurrent visual hallucinations, REM sleep behavior disorder, and parkinsonism (including bradykinesia, rigidity, and tremor) [5]. Point and period prevalence estimates of DLB range from 0.02 to 63.5 per 1000 persons [6], with considerable variability likely related to underdiagnosis of the disorder [7, 8].

As PD and DLB are diagnosed after there has been significant degeneration (e.g. PD results in 50–70% loss of neurons in the substantia nigra before clinical diagnosis occurs [9]), it is crucial to develop diagnostic tools and interventions that can be used early in the disease course. Idiopathic REM sleep behavior disorder (iRBD) is one of the earliest and most specific prodromal indicators of Lewy body diseases [10–12] with one in three idiopathic iRBD patients converting to a Lewy body disease within 5 years, 82.4% at 10.5 years, and 96.6% at 14 years. Of the converters, 44% will convert to PD and 25% to dementia with Lewy bodies [13]. Therefore, iRBD cohorts provide a unique opportunity to identify early disease mechanisms and develop neuroprotective interventions for Lewy body diseases.

The phenotypic presentation of Lewy body diseases largely depends on the location of the pathological initiation and progression [14–16]. While the clinical phenotypes of Lewy body diseases differ, the aggregation of alpha-synuclein (α-syn) is a defining feature [17, 18]. Through careful assessment of the propagation pattern of PD pathology, Braak and colleagues proposed that PD may be caused by an intestinal pathogen [19]. Several reviews have discussed the evidence to suggest that the gut plays an important role in the initiation or progression of pathological processes [20–24], though many questions remain regarding the mechanisms by which gut dysfunction leads to the development of Lewy body diseases. In the current review, we discuss two main hypotheses by which gastrointestinal dysfunction contributes to pathogenesis or disease progression. The first builds on Braak’s initial theory and posits that Lewy body diseases are caused by α-syn aggregation in the gut which travels in a prion-like fashion to the central nervous system (CNS). The second, more recent hypothesis emphasizes chronic intestinal proinflammatory processes as key mechanisms [24]. These theories are not mutually exclusive as there are likely interactions between immune responses and α-syn at all stages of the disease process. How early in the disease course a proinflammatory response versus α-syn aggregation occurs is an area of controversy and active investigation. Notably, both of these hypotheses emphasize dysbiosis (an imbalance in the microbiota) and intestinal permeability (translocation of lumen products across the gut wall, also referred to as “leaky gut”) as key mechanisms in the initiation and progression of pathophysiology in Lewy body diseases.

## Gastrointestinal dysfunction

Historically, PD research had focused on the motor symptoms of the disorder, though it is increasingly appreciated that gastrointestinal dysfunction, most commonly constipation, is one of the earliest prodromal symptoms of PD [25]. PD patients are three times more likely to experience constipation [26]. Constipation can precede motor deficits of PD by decades [27, 28] and is associated with worse outcomes, including earlier onset of dementia [29, 30]. In addition, patients may also experience sialorrhea and dysphagia, gastroparesis, and small intestinal bacterial overgrowth (SIBO), demonstrating pan-gut involvement [31, 32]. LBD patients experience similar, if not more severe gastrointestinal symptoms, with evidence that gastric emptying is slower in DLB patients relative to PD [33], though there is currently limited examination in DLB. Of note, objective colonic dysfunction is far more prevalent than subjective constipation in PD, highlighting the need to incorporate objective assessments to detect gastrointestinal dysfunction in Lewy body diseases [34].

SIBO was historically viewed as a cause of malabsorption and required invasive aspiration to obtain cultures of jejunal aspirate to diagnose [35]. Over time, it was recognized that intestinal bacteria were the sole source of certain gases, such as hydrogen and methane, that could be detected in exhaled breath. This was leveraged to develop glucose and lactulose breath tests for SIBO [36]. Using this approach, ~ ½ of PD patients test positive for SIBO [37, 38] and the occurrence of SIBO was associated with more severe motor fluctuations [38, 39]. SIBO eradication led to a significant improvement in patients OFF time and delayed ON episodes each day, though there is a high rate of SIBO relapse at 6 months (43%) [38]. SIBO was not directly related to small bowel transit delays [40] nor associated with worse gastrointestinal symptoms, but independently predicted worse motor function [41]. Gastrointestinal disorders that exhibit increased rates of SIBO, such as ulcerative colitis, Crohn’s disease, and irritable bowel syndrome are associated with an increased risk of PD [42, 43], suggesting SIBO may increase risk of PD or may play a role in the pathogenesis of Lewy body diseases. Evaluation of SIBO has been primarily conducted in more advanced patients. Given potential improvement in motor functioning, it is important to evaluate SIBO within prodromal, early stage PD, and DLB cohorts to understand the impact across phenotypes. For a detailed review of gastrointestinal symptoms in PD, refer to [26, 27, 32].

## Pathological processes

Lewy bodies and Lewy neurites are the defining neuropathological characteristics of PD and DLB [44–46]. Point mutations in the gene encoding α-syn (*SNCA*) were found to be pathogenic for familial forms of PD [47], which led to the subsequent discovery that α-syn is the principal component of Lewy bodies [17]. α-syn in its normal form is found within the presynaptic regions of neurons, either unfolded or contained in alpha-helical membrane-bound forms. Aggregation refers to the process by which α-syn becomes partially folded and aggregates to form oligomers, protofibrils, fibrils, and mature Lewy bodies [17, 48, 49]. It is unclear whether these variants of protein structure reflect distinct pathologies or a continuum of conformations reflecting the different stages of Lewy body diseases. Further, it is likely that in addition to a “triggering” event that initiates α-syn aggregation, it is likely that additional mechanisms are necessary to facilitate (allowing the disease to spread to the CNS) and aggravate the disease process (promote neurodegeneration beyond the basal ganglia) [50].

As noted, Braak and colleagues proposed that PD may be caused by an intestinal pathogen which travels through enteric neurons before entering the CNS via the vagus nerve [19]. Increasing evidence supports this hypothesis, including evidence of α-syn pathology in the intestinal wall examined both antemortem [51, 52] and postmortem [53, 54]. The α-syn deposits have been observed up to 20 years prior to a PD diagnosis [55]. Examination of colonic biopsies in iRBD cohorts has also demonstrated the presence of α-syn in these prodromal cohorts [56]. Recent work has demonstrated that gut microbes are able to promote α-syn-mediated motor deficits, brain pathology, and neuroinflammation in a mouse model of PD [57]. This evidence has led to an interest in identifying how intestinal dysbiosis and intestinal permeability, may play a mechanistic role in the initiation of α-syn aggregation at the level of the gut.

However, neuropathological studies using large cohorts have failed to find evidence of cases in which α-syn pathology is present in the peripheral nervous system in the absence of CNS pathology [58, 59]. Alternatively, intestinal inflammation may be the driver of disease pathogenesis in the periphery [24, 60]. It is well established that neuroinflammation is present in PD, though it was initially considered a response to α-syn aggregation rather than a primary mechanism of disease initiation [61]. More recently, it is hypothesized that intestinal dysbiosis and inflammation are the earliest disease processes that initiate both innate and adaptive immune system activation [60]. Specifically, a toxic trigger or changes in microbiota may contribute to dysbiosis and facilitate a proinflammatory environment. These processes increase intestinal permeability, resulting in increased levels of circulating proinflammatory cytokines, innate and adaptive immune cell activation, increased blood–brain barrier permeability, peripheral cell infiltration of the central nervous system, and neuroinflammation. While this process upregulates native α-syn expression which could potentially trigger its aggregation in the peripheral nervous system, the mechanistic emphasis is on the inflammatory processes.

## Clinical phenotypes

Clinically, the distinction between PD and DLB is made based on the temporal onset of motor versus cognitive symptoms (e.g., motor symptoms occur first = PD; cognitive symptoms occur first = DLB). However, both PD (including PDD) and DLB patients can exhibit similar clinical symptoms as the disease progresses. For instance, while hallucinations and RBD are diagnostic criteria for DLB, these same symptoms are present in 40–50% [62, 63] and 39–50% [64, 65] of PD patients, respectively. Additionally, cognitive impairment is common in PD, with up to 83% of patients exhibiting dementia after 20 years [66]. It is well established that the motor and cognitive symptoms of the disorders are closely linked to α-syn aggregation suggesting that disease mechanisms are the same across the disorders [1, 67], with differences in the clinical presentation related to the anatomical location of disease initiation and progression [16, 68].

Revisions of Braak’s original pathological staging have addressed some of the heterogeneity in pathological progression within the brain that leads to PDD versus DLB [69]. Given the evidence for a possible gut origin in some patients, a recent revision to Braak’s pathological staging has proposed two distinct paths of pathological initiation and progression. Either α-syn aggregation originates in the CNS (brain first) or the peripheral nervous system (body first) [15, 70], with the spread of pathology in a bidirectional manner. The neural connectome thus plays a crucial role in determining how α-syn propagates through the nervous system [16]. This theory is particularly useful for understanding the heterogeneity in phenotypes across Lewy body diseases. For example, in the body-first subtype, the α-syn pathology presumably originates in the enteric or autonomic nervous system and spreads to the CNS via the vagus and sympathetic connectome. These patients develop iRBD in the prodomal phase, have more autonomic and gastrointestinal symptoms, significant hyposmia, and faster motor and non-motor symptoms progression. This clinical presentation also largely overlaps with many symptoms observed in DLB, such as iRBD, autonomic dysfunction, and more severe cognitive dysfunction. Given the common neuropathology (α-syn aggregation) across these disorders and overlap in symptoms, this highlights the need to evaluate gastrointestinal mechanisms of pathogenesis across Lewy body diseases, as disease mechanisms likely span across diagnostic categories and may better account for the heterogeneity of clinical phenotypes.

## Dysbiosis

Microorganisms that live inside and on humans, referred to as microbiota, have symbiotic relationships with the human host. However, dysbiosis can lead to many disease processes, such as SIBO, Crohn’s disease, and inflammatory bowel disease [71, 72], with more recent evidence supporting the role of microbiota in neurodegenerative conditions [73]. Initial estimates suggested a ratio of 10:1 between bacteria and human cells, with more recent evidence suggesting a 1:1 ratio with approximately 3.9 × 10^13^ bacteria in/on the human body [74]. The sheer number of microbiota leads to complex dynamics that raises considerable challenges in evaluating the patterns of microbiota variation and the impact of dysbiosis.

The most common methodological approach includes the use of 16S rRNA sequencing of either fecal or intestinal samples. The 16S rRNA gene is conserved in all bacteria allowing for taxonomic identification. Despite over 25 studies using this approach in fecal samples in PD, there is considerable variability among findings and over 100 differently abundant taxa between PD patients and controls [75, 76]. A meta-analysis and pooled re-analysis of ten available studies that used 16S rRNA-gene amplicon sequencing indicated that the gut microbiome significantly differs between PD patients and controls, though the interstudy variability was the main factor driving bacterial community structures and only 1% of the total variance was accounted for by group status [76]. When inconsistencies across studies (country of origin, sampling protocols, sample storage, DNA extractions, and sequencing strategies) were accounted for, PD patients exhibited a reduction of the genera *Roseburia, Fusicategnibacter, Blautia, Anaerostipes* (Lachnospiraceae family), and *Faecalibacterium* (Ruminococcaceae family) in addition to enrichment of the *Lactobacillus, Akkermansia, and Bifidobacterium* genera. No significant differences in enterotypes were observed. Similar reports were observed in a recent review [75]. Increased Akkermansia (14 out of 30 studies)*,* followed by *Lactobacillus* (7 out of 30 studies)*,* and *Bifidobacterium* (10 out of 30 studies) whereas decreased abundance of *Roseburia* (8 out of 30), *Faecalibacterium* (8 out of 30 studies), and *Blautia* (7 out of ten studies) were most consistently observed.

There are numerous reasons for variability across studies, including collection and assaying methods, with poor control of confounding factors, such as antibiotic use in early life or previous gastrointestinal infections [77]. There is an enormous amount of individual and geographic variability that raises challenges when attempting to quantify group differences in the microbiome [78, 79]. Finally, the majority of prior evaluations of the microbiome quantify microbial taxa and metabolic pathways as fractions of the sample sequence generated by each analysis, rather than disease-associated imbalances that may occur [77, 80].

## Increases in rare species of microbiota

While the prior studies often detect changes in the more prevalent microbiota species, it can be challenging to detect rare species that, when they increase in number, can exert adverse biological effects. As part of the pooled re-analysis by Romano et al. [76] discussed above, microbial alpha-diversity and abundances of rare taxa were significantly increased in PD relative to control samples. This suggests a reduction in dominant species and an increase in rare/low abundant ones. When commensal bacteria increase in number to exert adverse biologic effects, they are known as pathobionts. For example, sulfate-reducing bacteria are rare members of the gut microbiome under normal conditions (a fraction of a percent) and help to support microbial fermentation by converting its metabolite, hydrogen, to hydrogen sulfide (H_2_S). However, when dysbiosis occurs, sulfate-reducing bacteria can increase in number (bloom in sulfate-reducing bacteria), becoming pathogenic as an increase can impair intestinal barrier and increase levels of potentially toxic H_2_S. The *Desulfovibrionaceae* family, the most prominent family of sulfate-reducing bacteria [81], is elevated in PD patients [82, 83] and the concentration of *Desulfovibrio* species correlates with the severity of PD [83]. As a consequence, PD patients may exhibit excess H_2_S. H_2_S can be beneficial as it acts as a gaseous neurotransmitter produced in small quantities by the host regulating a number of body functions including gastrointestinal, neuronal, cardiovascular, endocrine, respiratory, renal, and hepatic systems [84]. However, elevated levels of H_2_S produced by a bloom in sulfate-reducing bacteria can become harmful to the host and is associated with gastrointestinal disorders such as ulcerative colitis, Crohn’s disease, and irritable bowel syndrome [84–86]. As noted, these disorders are linked with an increased risk of PD [42, 43].

## Consequences of dysbiosis

### Reduction in short-chain fatty acids

*Roseburia, Fusicategnibacter, Blautia,* and *Anaerostipes* are butyrate producers, a short-chain fatty acid (SCFA). SCFAs are produced by the fermentation of dietary fiber by microbiota and are exclusively produced in the intestine. They exhibit anti-oxidant and anti-inflammatory processes and regulate the expression of tight junction proteins, which can impact intestinal barrier integrity [87]. Absolute concentrations of SCFAs are significantly reduced in human PD fecal samples, including butyrate, acetate, and propionate [88]. A decrease in fecal levels of butyrate has been associated with intestinal inflammation in PD patients [89]. Examination of plasma SCFAs suggested opposite effects, with increased SCFAs in PD relative to a matched cohort [90]. Taken together, the observed reductions in *Roseburia, Fusicategnibacter, Blautia,* and *Anaerostipes* may contribute to proinflammatory shifts in microbiota composition in PD.

### Increase in self-peptides

*Lactobacillus, Akkermansia, and Bifidobacterium* genera are typically considered to be beneficial bacteria, suggesting either a role in PD or simply that these bacteria are well adapted to thrive in the context of dysbiosis. However, an enrichment of *Akkermansia* has been observed in multiple sclerosis patients and is associated with a proinflammatory response [91–93]. Recent evidence suggests that peptides produced by *Akkermansia* may interact with autoreactive T cells in multiple sclerosis [94]. Specifically, the human leukocyte antigen (HLA)-DR15 haplotype has been associated with the pathogenesis of multiple sclerosis [95] via the abundant production of HLA-DR-derived self-peptides. *Akkermansia* may mimic these peptides ultimately sensitizing activated CD4 T cells in the periphery which leads to pathogenic autoreactive T cells in the brain [94]. Given the increases in *Akkermansia*, it is possible that similar mechanisms may occur in Lewy body diseases.

### Increase in lipopolysaccharide

The observed increase in the density of Gram-negative bacterial strains (including *Akkermansia* and *Desulfovibrio*) in PD fecal samples [96, 97] corresponds to an increase in endotoxin lipopolysaccharide (LPS) content, as LPS is a cell wall component of Gram-negative bacteria [98]. LPS is a known endotoxin that can lead to increased intestinal permeability [99–101]. Plasma and serum lipopolysaccharide-binding protein is reduced in PD [102–104]. Lipopolysaccharide-binding protein increases when LPS is elevated acutely [105, 106], but decreased when there has been chronic exposure, suggesting PD patients experience prolonged elevated LPS. LPS in this context may directly contribute to the initiation of α-synuclein at the level of the gut [107] as it has demonstrated the ability to promote α-synuclein aggregation via the formation of intermediate nucleating species [108–110]. Alternately, LPS may lead to systemic inflammatory processes in the context of increased intestinal permeability that contribute to disease pathogenesis, as it is a potent stimulator of microglial activation and has been associated with degeneration of the neurons in the SNc and motor deficits [111]. Specifically, LPS activates toll-like receptors initiating an innate immune response stimulating the production of inflammatory cytokines (such as IL-1, TNF-α, and IL-6) and reactive oxygen species [112, 113].

### Increase in H_2_S

Several pathological processes may occur when *Desulfovibrio* colonizes the intestine, including increased generation of H_2_S in amounts that exceed detoxification capacity, increased inflammatory responses, and increased intestinal permeability (leaky gut) [114, 115]. *Desulfovibrio* also has the ability to produce magnetite (Fe_3_O_4_) [116], which may accelerate α-syn aggregation [117]. A recent model has proposed that the increase in H_2_S concentrations causes leaky membrane resulting in the release of cytochrome c from the mitochondria and an increase in cytosolic iron levels. This, in combination with magnetite nanoparticles originating from *Desulfovibrio* species may result in α-syn aggregation via production of reactive oxygen species [118].

### Increase in curli protein

Preclinical studies have suggested that the amyloid protein, curli, produced by *Escherichia coli* (*E. coli*), may play an important role in the initiation of α-syn initiation. Specifically, in the context of increased proteobacteria (Gram-negative bacteria), there will be more *E. coli,* a bacterium that secretes curli. Rats exposed to curli-producing bacteria (*E. coli*) displayed increased neuronal α-syn deposition in both the gut and brain as well as enhanced microgliosis and astrogliosis. Together, this suggests that curli, a gut bacterial amyloid protein, may trigger the initiation of α-syn aggregation [119]. This role of curli was supported by the finding that curli expression was required for *E. coli* to exacerbate α-syn-induced behavioral deficits. In addition, oral treatment of mice with a gut-restricted inhibitor of amyloid prevented curli-mediated acceleration of PD-like pathology and behavioral abnormalities [120]. A separate line of work showed that LPS may contribute to the pathophysiology by accelerating the synthesis of curli fibrils [121].

### Increased intestinal permeability

The intestinal barrier, which consists of physical (mucus, tight junction proteins), and chemical (anti-microbial peptides) components, shields the intestine from the contents of the lumen. Barrier integrity is reliant on the tight junctions, which include claudins, occludin, zonula occludens, adheren junctions, desmosomes, and gap junctions [122]. Damage to the barrier can allow α-syn, microbes, environmental toxins, or other luminal contents to gain access to the submucosal neuronal tissue or systemic circulation. Several studies have demonstrated that PD patients exhibit intestinal barrier dysfunction [89, 123–125], referred to as ‘leaky gut’ that is associated with microbial translocation across the intestinal mucosa. Factors resulting from dysbiosis, such as increases in LPS [99–101] and *Desulfovibrio spp.* [126] also lead to leaky gut. Recent work has demonstrated that *Desulfovibrio spp.* induced intestinal permeability via the snail pathway [126]. Snail is a transcription factor associated with increased intestinal permeability [127–129] via negatively regulating tight junctions [130, 131].

Leaky gut may contribute to the entry of known modulators of α-syn aggregation, such as curli, H_2_S, and LPS, into the systemic circulation and then beyond, to end organs such as the brain. For example, increased intestinal permeability and *E. coli* staining correlated with α-syn staining, supporting the contribution of leaky gut and this bacteria to α-syn aggregation [123]. Additional evidence has also demonstrated associations between intestinal permeability and pathological α-syn aggregation [132].

### Inflammation

As we discussed above, many of the observations thus far suggest the observed patterns of altered microbiota composition facilitate a proinflammatory shift in PD, including a reduction in SCFAs and an increase in immune system activating peptides. LPS activates toll-like receptors that initiate an innate immune response and the production of inflammatory cytokines. The production of H_2_S functions as an endogenous regulator of the immune system [133] and thus an increase in H_2_S secondary to a bloom in sulfate-reducing bacteria could contribute to a proinflammatory response. Aspects of these processes can occur with intact intestinal barrier, though an increase in intestinal permeability facilitates the release of lumen products contributing to a systemic inflammatory response driven by the innate and adaptive immune systems [134, 135], which may play a key role in sustaining and exacerbating α-syn aggregation [24].

#### Pathways to the brain

**Fig. 1:**
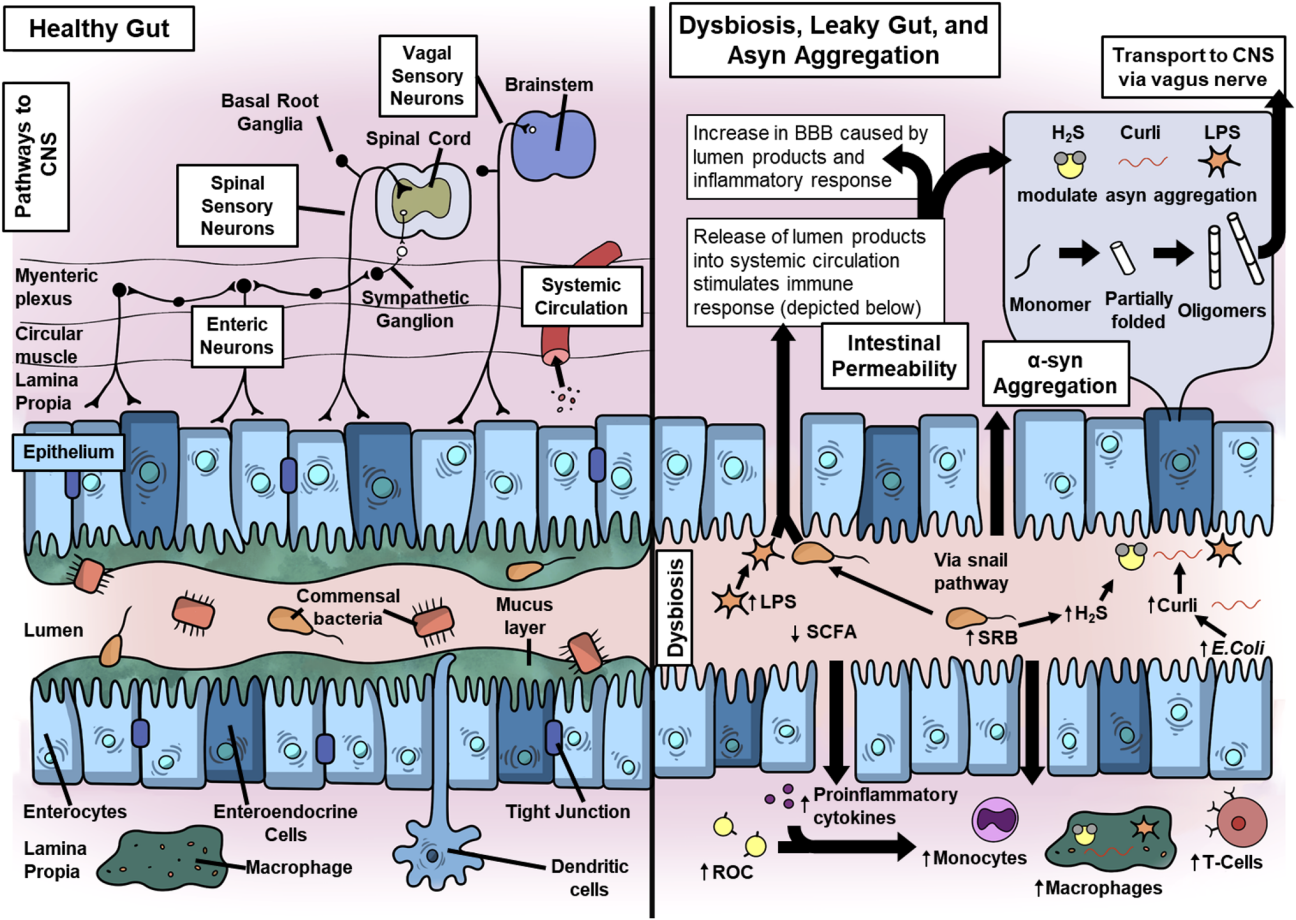
Proposed mechanisms of early disease processes in Lewy body diseases. Left panel: healthy gut. In a healthy gut, the commensal microbes, epithelium and immune cells maintain an equilibrium. Right panel: dysbiosis includes decrease in *Roseburia, Fusicategnibacter, Blautia,* and *Anaerostipes*, leading to a reduction in the production of short-chain fatty acids (SCFAs). An increase in *Lactobacillus, Akkermansia,* and *Bifidobacterium* is considered beneficial bacteria, though Akkermansia may produce peptides that mimic self-peptides that sensitize T cells. An increase in Gram-negative bacteria strains increase lipopolysaccharide (LPS), an endotoxin that can damage the intestinal barrier and initiate inflammatory processes. A bloom in sulfate-reducing bacteria (SRB) increases hydrogen sulfide (H_2_S) production, which may facilitate α-syn aggregation, increase intestinal permeability, and initiate an inflammatory response. *E. coli* increases levels of curli protein, which has been implicated as a modulator of α-syn aggregation. α-Syn may aggregate locally via these mechanisms within the enteroendocrine cells and travel via the vagus nerve to the brainstem. Additionally, the activation of both innate and adaptive inflammatory responses increases the circulating proinflammatory cytokines, reactive oxygen species (ROC), monocytes, macrophages, and T cells. The release of lumen products and triggering of inflammatory processes can damage the blood–brain barrier (BBB), facilitating infiltration of the pathogenic processes into the central nervous system (CNS)

There are multiple paths for bidirectional gut–brain communications (Fig. 1), involving neural pathways as well as immune and endocrine mechanisms [136]. The vagal and spinal sensory neurons receive signals within the lamina propria and are directly connected to the brainstem and spinal cord, respectively. Additional pathways connect the enteric nervous system with CNS. For example, signaling from intrinsic primary afferent neurons are conveyed by intestinofugal nerves to the spinal cord via sympathetic ganglia.

Braak’s initial theory posited that α-syn pathology has the ability to spread from the gastrointestinal tract to the brain via the vagus nerve [19]. Epidemiological evidence has demonstrated that a full truncal vagotomy decreases the risk of PD [137, 138]. Animal models have also shown that recombinant α-syn injected into the intestinal wall could be transported via the vagal nerve to reach the dorsal motor nucleus in the brainstem [139]. Additionally, the injection of preformed α-syn fibrils into the muscle layers of the pylorus and duodenum, which is densely innervated by the vagus nerve, leads to their propagation to the CNS following a path similar to that characterized by Braak [140]. The potential cellular mechanisms of α-syn propagation have been reviewed elsewhere [141], with increasing evidence supporting the notion that it propagates in a prion-like fashion [142], which would provide a mechanism by which α-syn aggregation in the gut could propagate to the CNS.

There are multiple potential sites within these pathways in which α-syn aggregation could initiate, including the enteric nervous system [143]. However, recent efforts have proposed that gut enteroendocrine cells may serve as sites for the initial emergence of pathogenic α-syn [144]. Enteroendocrine cells are chemosensory cells dispersed throughout the mucosal lining of the intestine and their apical surface is open to the lumen of the intestine. Historically, they were viewed as hormone-producing cells, but subsequent observations have demonstrated that they are electrically excitable, possess many neuronal features, and can communicate directly with the nervous system [145, 146]. Additionally, α-syn is expressed by the enteroendocrine cells, both in the small and large intestine, highlighting that they may serve as loci for the initial pathological α-syn aggregation [144].

This work largely supports the notion that it is physiologically feasible for α-syn aggregation to begin in the gut and travel to the CNS. However, as noted, there has been limited evidence using immunohistochemical staining of cases in which α-syn pathology was observed in the gut in the absence of CNS pathology [58, 59]. Alternatively, dysbiosis and intestinal permeability may lead to an increase in the entry of lumen products (including LPS, H_2_S, and curli proteins) as well as cytokines and immune cells into systemic circulation [147, 148]. Rather than initiating α-syn aggregation in the periphery, these processes may contribute to disease mechanisms by impacting the blood–brain barrier (BBB) and facilitating a prolonged immune response. This would facilitate peripheral cell infiltration across the BBB which contributes to neuroinflammation.

#### Increased blood–brain barrier permeability

The BBB is a physiological barrier that protects the brain from unwanted molecules in the blood. Similar to the intestinal barrier, the BBB leakage is driven by damage to endothelial tight junctions, which include occludin, claudins, zonula occludens, and adheren junctions, though the BBB has added complexity given the sensitivity of the brain to toxins and pathogens [149]. In addition to changes in tight junctions, BBB permeability can also be altered by damage to endothelial cells or astrocytes as well as degradation of extracellular matrix components. Disruption of the BBB likely plays an important role across neurodegenerative conditions [150], with emerging evidence that permeability of the BBB is increased in PD [151, 152]. Specifically, an increased ratio of cerebrospinal fluid albumin to serum albumin was observed in PD [153]. Thinning and fragmentation of tight junction proteins was observed in postmortem immunofluorescence staining evaluations of PD cases [154]. PET imaging has also indicated reduced P-glycopreotein 1 activity, suggestive of BBB dysfunction, in the midbrain in PD patients [155].

The gut microbiota can regulate the BBB via several potential mechanisms [156], including the direct impact of intestinal microbial metabolites such as LPS [157, 158], immune and endocrine responses [159], or upregulation of α-syn [160]. In terms of the consequences of dysbiosis discussed above, there are several potential mechanisms by which dysbiosis and intestinal permeability may lead to increased BBB in PD. Specifically, LPS has been used to study the impact of systemic inflammation on BBB function, indicating potential BBB dysfunction in 60% of studies [161], with BBB change observed more consistently in mice versus rats. These studies, however, typically use septic doses of LPS, which limits the generalizability to Lewy body diseases. While α-syn in its non-pathologic form can travel bi-directionally across the BBB, transportation is enhanced in the presence of LPS [162, 163], suggesting potential upregulation of α-syn in the brain. Additionally, increased *desulfovibrio spp.* can induce leaky gut via the activation of the snail pathway. This same pathway has also been found to disrupt BBB by impacting integrity of tight junctions [164].

Systemic inflammation induced by dysbiosis and intestinal permeability may also increase BBB permeability. For example, systemic inflammation induces migration of microglia to the cerebral vasculature to maintain BBB integrity by expressing tight-junction proteins and connecting with the endothelial cells. However, during sustained inflammation, microglia phagocytose the astrocytic end-feet of the BBB, impairing the BBB function [165]. Given the consistent finding of activated microglia in the postmortem brains of PD patients [166, 167], this would suggest that systemic inflammation may be driving these processes. Herein we focus our review on the potential mechanisms driven by gastrointestinal factors we have identified, however, for detailed review of immune dysfunction and neuroinflammation, please refer to [24, 60].

#### REM sleep behavior disorder

As noted above, iRBD is a prodrome of Lewy body diseases, with up to 96% of iRBD patients converting to a synucleinopathy, the majority would be diagnosed with either PD or dementia with Lewy bodies. In the “body first” Lewy body disease phenotype mentioned above, the pathology would theoretically progress from the dorsal motor nerve of the vagus to first impact the locus coeruleus [16], which is inferior to the substantia nigra and associated with the development of iRBD [168, 169]. As the disease progresses to the substantia nigra or broader regions, individuals may begin to exhibit symptoms consistent with either PD or LBD.

Self-reported gastrointestinal symptoms are elevated in iRBD cohorts relative to healthy controls, with significantly greater endorsement of constipation and straining for defecation [170, 171]. Total gastrointestinal transit time, colonic volume, and 3D-Transit colonic transit time were significantly increased in an iRBD cohort relative to controls, though not to the extent observed in medicated PD patients [172]. iRBD patients exhibit a microbiome similar to that seen in patients with PD [173] and colonic biopsies in iRBD cohorts showed the presence of α-syn [56], though SCFA were not reduced in iRBD as in PD [174].

Additionally, interleukin-10 levels are upregulated in iRBD relative to controls [175] in addition to tumor necrosis factor-α levels, which were found to predict phenoconversion to an α-synucleinopathy [176]. Increased microglial activation was detected by PET in the substantia nigra in addition to reduced dopaminergic function in the putamen [177]. iRBD patients’ blood monocytic cells showed increased expression of CD11b and decreased expression of HLA-DR. iRBD patients had increased classical monocytes and mature natural killer cells. The levels of expression of toll-like receptor 4 on blood monocytes was correlated with the nigral immune activation measured with PET [178].

Taken together, early gastrointestinal symptoms, such as constipation and dysbiosis, as well as systemic inflammation, are present in the prodromal stages of LBDs, however, there is a great need to understand the earliest changes, identify potential mechanisms, and whether these predict phenoconversion.

#### Early detection of pathological changes and targets for intervention

There are currently no disease-modifying treatments for Lewy body diseases, largely due to the lack of mechanistic understanding of disease pathogenesis and the challenges associated with targeting α-syn aggregates [179]. While there are numerous research questions to answer regarding the mechanisms of gastrointestinal dysfunction and the pathogenesis of Lewy body disease, there are several potential treatments that are readily available spanning antibiotics, probiotics, and fecal microbiota transplantation [180]. For example, Rifaximin is a broad-range, gastrointestinal-specific antibiotic used to treat SIBO, which improves motor symptoms [38]. Relatedly, numerous studies have evaluated the impact of probiotics on constipation symptoms in PD [181], with evidence of a reduced MDS-UPDRS total score [182]. Based on the emerging research presented herein, targeting specific bacteria offer intriguing possibilities.

Additionally, a major limitation of current PD medications is that they lose efficacy over time, with recent evidence that gut microbiota has been found to moderate the metabolism of Parkinson’s medication [183–185]. While not disease modifying, targeting these bacteria may significantly improve efficacy of PD medications [186]. Evolving evidence support PD and Lewy body diseases more generally as both central and peripheral diseases. Targeting the pathophysiology taking place in the gut offers exciting opportunities for early intervention. Could targeting dysbiosis or intestinal permeability *prior* to the development of α-syn aggregation be an effective way of forestalling Lewy body diseases before the disease is clinically diagnosable?
